# Experimental Validation of a Cardiac Simulator for *in vitro* Evaluation of Prosthetic Heart Valves

**DOI:** 10.5935/1678-9741.20160041

**Published:** 2016

**Authors:** Ovandir Bazan, Jayme Pinto Ortiz

**Affiliations:** 1 Department of Mechanical Engineering, Escola Politécnica of the University of São Paulo (EPUSP), Brazil

**Keywords:** Models, cardiovascular, Heart valve prosthesis, Heart rate

## Abstract

**Objective:**

This work describes the experimental validation of a cardiac simulator for three heart rates (60, 80 and 100 beats per minute), under physiological conditions, as a suitable environment for prosthetic heart valves testing in the mitral or aortic position.

**Methods:**

In the experiment, an aortic bileaflet mechanical valve and a mitral bioprosthesis were employed in the left ventricular model. A test fluid of 47.6% by volume of glycerin solution in water at 36.5ºC was used as blood analogue fluid. A supervisory control and data acquisition system implemented previously in LabVIEW was applied to induce the ventricular operation and to acquire the ventricular signals. The parameters of the left ventricular model operation were based on *in vivo* and in vitro data. The waves of ventricular and systemic pressures, aortic flow, stroke volume, among others, were acquired while manual adjustments in the arterial impedance model were also established.

**Results:**

The acquired waves showed good results concerning some *in vivo* data and requirements from the ISO 5840 standard.

**Conclusion:**

The experimental validation was performed, allowing, in future studies, characterizing the hydrodynamic performance of prosthetic heart valves.

**Table:** 

Abbreviations, acronyms & symbols	
AoF	= Flow rate in the aortic root
AoP	= Aortic pressure
AV	= Aortic valve
bpm	= Beats per minute
CO	= Cardiac output
DiaD	= Diastole duration
DynVisc	= Dynamic viscosity of blood
EDV	= End-diastolic volume
EPL	= Ejection phase length
EPUSP	= Escola Politécnica of the University of São Paulo
ESV	= End-systolic volume
FDA	= Food and Drug Administration
HR	= Heart rate
LDA	= Laser Doppler anemometry
LVDP	= Left ventricular diastolic pressure
LVP	= Left ventricular pressure
LVV	= Left ventricular volume
MV	= Mitral valve
PIV	= Particle image velocimetry
PxV	= Left ventricular pressure *versus* left ventricular volume
SV	= Stroke volume
SysD	= Systole duration

## INTRODUCTION

For almost sixty years, pulse duplicator systems or cardiac simulators (*i.e.*, left ventricular models) have been designed to replicate the pressure and flow waves according to the human cardiovascular physiology^[1-8]^. Cardiac simulators are required for experimental evaluation of ventricular assist devices^[9-11]^ and to allow the hydrodynamic performance testing of prosthetic heart valves^[12-16]^.

Although the main goal of most cardiac simulators is to mimic left ventricular and systemic circulation, pulse duplicators conception and control loop design can be different according to the experimental purpose. Once the evaluation of left ventricular assist devices is performed, for instance, based on adaptive estimation of the aortic pressure and suitable response regarding the left ventricular contractility variation^[10,17-20]^, the operation of cardiac simulators in this case is designed to an automatic variation of cardiovascular parameters (according to the Frank-Starling mechanism), requiring a full closed-loop control. However, in terms of prosthetic heart valves testing, the cardiac simulators are used only to conduct cyclic operation with good repeatability, in some predicted ventricular conditions^[12]^. In this case, the cardiac simulator control system can be simplified, but the ventricular model is expected to mimic some anatomical and flow characteristics of the human heart^[21,22]^. Besides, optical accesses are needed in the ventricular model to apply laser velocimetry techniques (*i.e.*, particle image velocimetry, or PIV, and laser Doppler anemometry, or LDA) in order to characterize the flow through the valves^[12,13,15,23]^. Pathological case studies also are possible in cardiac simulators, although a proper validation is required^[7,9,20,24]^. In physiological and pathophysiological conditions, different levels of stenosis can be simulated^[25]^.

This work describes the experimental validation of a left heart simulator at Escola Politécnica of the University of São Paulo (EPUSP), under physiological conditions and using normal prosthetic valves (with no valve thickening nor simulated stenosis), working at three heart rates (HR): 60, 80 and 100 bpm (beats per minute). *in vivo* data were used to analyze the responses from the cardiac simulator for each HR. This validation will allow, in future works, the hydrodynamic testing of mitral and aortic prosthetic valves based on laser velocimetry techniques.

## METHODS

### Parameters of Blood Rheology and Cardiac Physiology

Ventricular parameters from a normal healthy person^[26-41]^ were examined in order to induce the proper operation for the cardiac simulator and to determine parameters of comparison, according to Table 1.

**Table 1:** Parameters for comparison.

	60 bpm		80 bpm		100 bpm	
Param.	Ref.	CS	Ref.	CS	Ref.	CS
SV [mL]	70	70	62.5	62.5	55	55
EDV [mL]	120	120	120	120	120	120
ESV [mL]	50	50	57.5	57.5	65	65
CO [L/min]	4.2	3.8	5	4.2	5.5	4.9
EPL [ms]	210	250	-	250	-	250
AoP [mmHg]	80-120	80-120	[-]-125	104-127	100-140	118-132
LVP [mmHg]	0-'20	4-125	-	2-'50	-	2-'65
LVDP [mmHg]	0-'6	4 - '0	-	2-'8	-	2-28
SysD [ms]	380	360	360	360	358	360
DiaD [ms]	6'8	640	390	390	242	240
DynVisc [mPas]	4	4	4	4	4	4
T [°C]	36.5	36.5±0.5	36.5	36.5±0.5	36.5	36.5±0.5

Hemodynamic parameters (Param.) of reference^[25-39]^ for the comparison with the cardiac simulator (CS) responses: ventricular stroke volume (SV), end-diastolic volume (EDV), end-systolic volume (ESV), cardiac output (CO), ejection phase length (EPL), aortic pressure (AoP), left ventricular pressure (LVP), left ventricular diastolic pressure (LVDP), systole duration (SysD), diastole duration (DiaD), and dynamic viscosity of blood (DynVisc) in the physiological temperature (T).

Concerning hematocrit of 40%, shear rate of 212 sec^-1^, when certainly blood behaves as a Newtonian fluid^[37]^, and at 36.5ºC, the dynamic viscosity of blood is approximately 4 mPas^[38]^. In the rest condition (60 bpm), the end-diastolic volume (EDV) can be considered 120 mL. Once the ventricular stroke volume (SV) is 70 mL, the end-systolic volume (ESV) varies around 50 mL^[26]^. The left ventricular pressure (LVP) rises up to 120 mmHg, while the aortic pressure (AoP) increases from 80 to 120 mmHg^[31]^. Left ventricular diastolic pressure (LVDP, related to the period of ventricular filling) begins with 0 mmHg and ends close to 16 mmHg^[40]^. The ejection phase length (EPL) is approximately 210 ms^[31]^ and systole duration is close to 380 ms^[27,28]^.

The normal human cardiac response to exercise includes an increase of both preload and contractility when the systolic blood pressure is expected to rise^[26,29]^. However, this tendency is attenuated by the arterial baroreceptor function and the diastolic pressure generally remains near resting condition. Furthermore, due to exertion, there is a decrease in systemic vascular resistance and the increase in mean AoP is normally much smaller than the increment in cardiac output (CO)^[30]^, which is achieved mainly by increasing HR (CO = SV* HR). As HR increases, a reduction in the duration of systole and diastole occurs. However, this reduction is much more pronounced in the diastole phase^[27,28]^. The hemodynamic parameters for 60, 80 and 100 bpm (Table 1) were established according to *in vivo*^[27,28,39-41]^ and in vitro^[9,10,15,19,22]^ data, assumptions based on laboratory values^[35]^, the ISO 5840 standard^[12]^ and simulated states^[36]^.

### Cardiac Simulator

The cardiac simulator was conceived according to the Food and Drug Administration (FDA) guidance concerning prosthetic heart valves^[16]^ and the ISO 5840:2010 standard^[12]^, in which cyclic operation and good repeatability are required for some predicted ventricular conditions (involving suitable adjusts of SV, ESV, HR, compliance and peripheral resistance, among others). Automatic and dynamic adaptations regarding changes in physiological parameters are not expected^[12,16]^.

Cardiac simulator is based on human left ventricle and systemic circulation^[42]^. A supervisory control and data acquisition system using LabVIEW 2011 (National Instruments Corp., Austin, TX, USA), described previously^[43]^, was applied for ventricular operation and to acquire the ventricular signals for each HR.

**Fig. 1:**
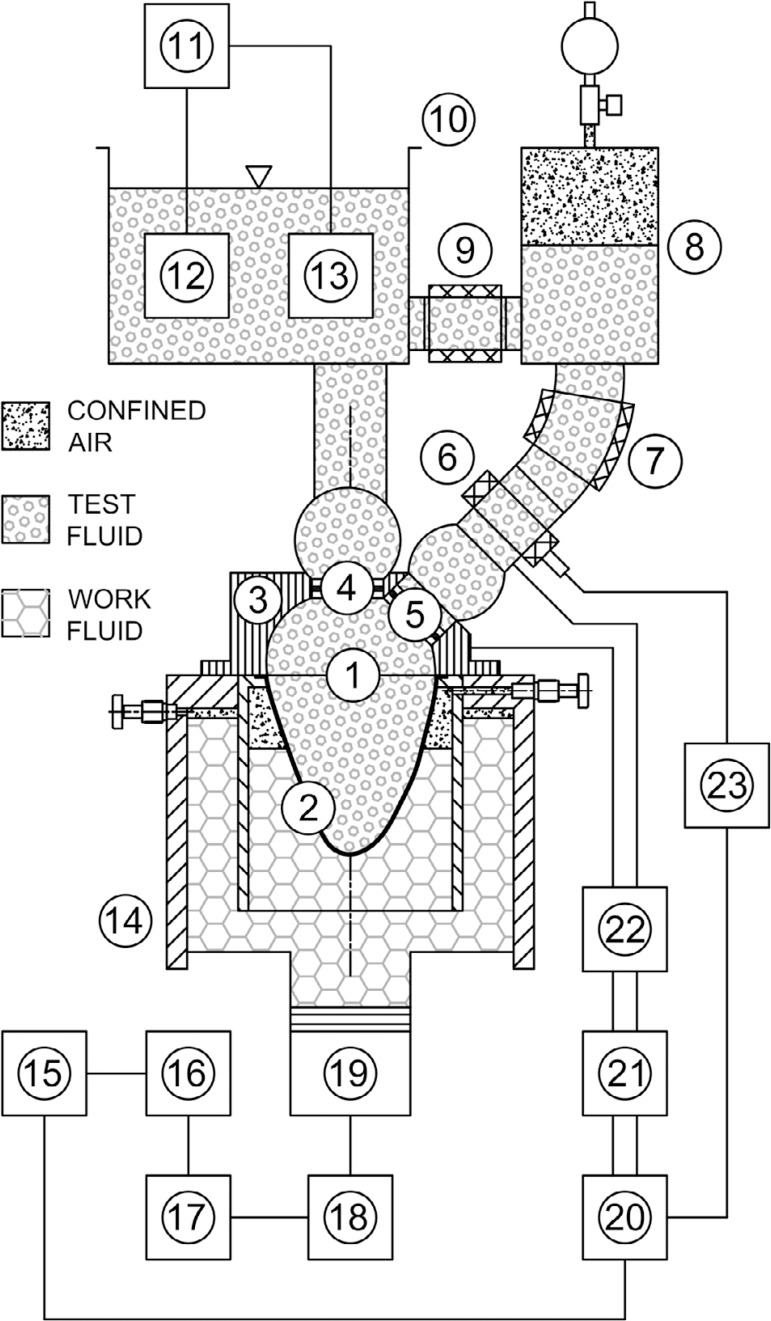
Schematic model of the cardiac simulator. Left ventricular model (1), flexible membrane (2), optical platform made of acrylic apt to laser velocimetry applications (3), mitral valve (4), aortic valve (5), flow probe (6), characteristic resistance (7), adjustable compliance (8), adjustable peripheral resistance (9), pre-atrial reservoir (10), digital thermostat (11), temperature sensor (12), heater (13), work fluid reservoir (14), microcomputer to run the supervisory control and data acquisition system (15), servomotor drive (16), servomotor (17), linear slide table (18), hydraulic cylinder (19), DAQ module (20), signal conditioners (21), invasive blood pressure transducers (22), and flowmeter (23).

Figure 1 shows the schematic model of the left heart and systemic circulation.

According to Figure 1, n. 20, a multifunction data acquisition (DAQ) module (NI USB-6212 BNC: 16-Bit, 400 kS/s, National Instruments Corp., Austin, TX, USA) was used connected to the instrumentation and the microcomputer running the LabVIEW. The instrumentation incorporated to the cardiac simulator includes two invasive blood pressure transducers (type 257365-BXSN, Braile Biomédica Ind. Com. e Rep. S.A., São José do Rio Preto, SP, Brazil, Figure 1, n. 22) supplied by two amplifier circuits with gain of 25.36 (as proposed in the AD620 datasheet, Analog Devices Inc., Norwood, MA, USA, Figure 1, n. 21), an electromagnetic flowmeter (model 501, Carolina Medical Electronics Inc., East Bend, NC, USA, Figure 1, n. 23), and a temperature control system (model TLZ11, Coelmatic Ltda, São Paulo, SP, Brazil, Figure 1, n. 11-13), which works independently from the DAQ module. The two invasive blood pressure transducers enable the LVP and AoP signals acquisition. The flow probe (300A series, Carolina Medical Electronics Inc., East Bend, NC, USA, Figure 1, n. 6) is located in order to acquire the signals of flow rate in the aortic root (AoF).

The left ventricular model (Figure 1, n. 1) is composed of two parts, a flexible membrane (Figure 1, n. 2) made of silicone, and the optical platform (Figure 1, n. 3), which enables the use of PIV and LDA techniques^[12]^. The operation of the left ventricular model is due to the flexible membrane deflection, besides the proper positioning of the aortic and mitral prosthesis to provide the correct flow direction. The linear slide table (Figure 1, n. 18) converts the rotational motion of the servomotor shaft into a linear displacement of a piston within the hydraulic cylinder (Figure 1, n. 19), connected to the work fluid reservoir (Figure 1, n. 14). Then, the shaft rotation induces volumetric changes into the work fluid reservoir. It allows controlling the work fluid pressures and, therefore, the flexible membrane deflection. Depending on the rotational direction of the servomotor shaft, it implies a ventricular inflow or ejection, according to the unidirectional flow provided by each prosthetic valve.

The left ventricular model (Figure 1, n. 1) was designed so that the geometry, size and valves positioning were similar to the natural left heart anatomy. The volumetric capacity of the ventricular chamber (up to 220 mL) was combined with the viability of laser velocimetry applications. The optical platform (Figure 1, n. 3) is completely exposed to the atmosphere^[42]^.

Through the DAQ module and LabVIEW, a pulse counter input was established to quantify the rotation of the servomotor shaft via an encoder. Every shaft revolution was discretized in 1,000 pulses. Thereby, the left ventricular model operation during cardiac cycles was referred to the servo motor encoder signals, allowing knowing the residual left ventricular volume (LVV) as a function of time, which is important to establish the LVP *versus* LVV diagram of the left ventricular model.

Supervisory control system allows modulating the servomotor drive parameters of the shaft rotation, such as velocity, acceleration, number of revolutions and waiting times. It allows the simulator to induce the SV, the HR and proper duration of systole and diastole, for instance. Indirectly, it also enables to establish the EDV and the ESV.

Arterial impedance (a Windkessel model based on three elements, Figure 1, n. 7-9) is not a function of a full closed-loop control system. Therefore, manual adjustments of compliance and resistances were required. Moreover, the test fluid level in the pre-atrial reservoir (Figure 1, n. 10) determines the atrial pressure^[3]^. Also, air volumes can be injected into the work fluid reservoir (Figure 1, n. 14 confined air) in order to adjust the sensitivity of the flexible membrane actuation.

### Experimental Procedure

**Fig. 2:**
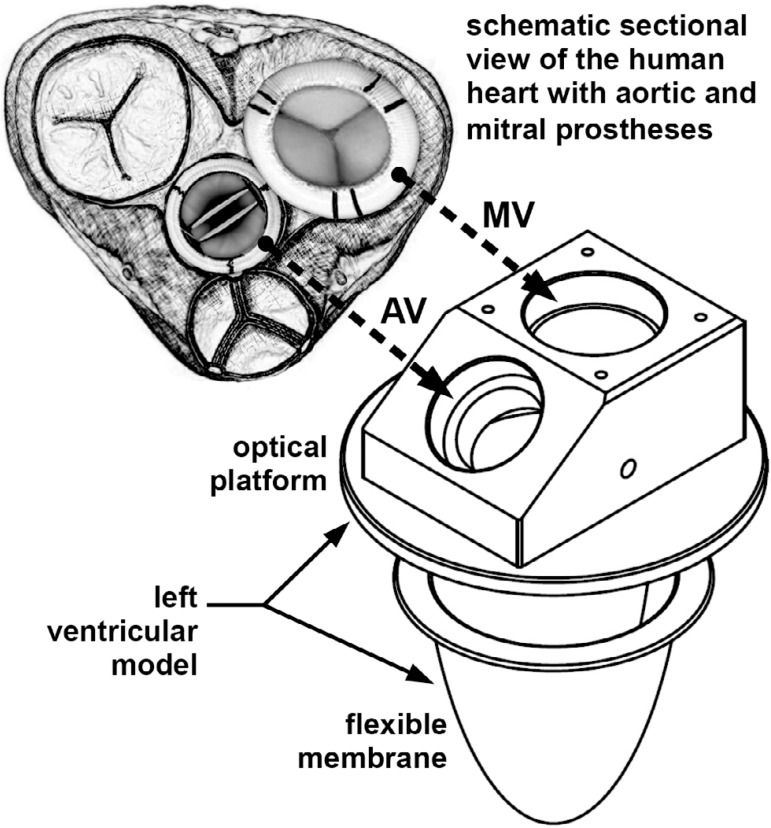
Assembly drawing of the prostheses in the left ventricular model. Prosthetic aortic valve (AV), and prosthetic mitral valve (MV).

An aortic bileaflet mechanical valve (CarboMedics Inc., Austin, TX, USA, 27 mm diameter) and a stented tricuspid mitral bioprosthesis of bovine pericardium (confidential information, 31 mm diameter) were used in the left ventricular model. The aortic and mitral valves were positioned as shown in Figure 2.

A glycerol-water mixture with 47.6% by volume of glycerin solution in water (with normal saline solution to allow the electromagnetic flowmeter operation) at 36.5±0.5ºC was used as a blood analogue fluid. It implies a dynamic viscosity of approximately 4 mPas^[44]^.

The supervisory control system was configured to send parameters to the servomotor driver fixing SV, ESV, HR, and DiaD, according to Table 1. The same SysD of 360 ms was fixed for the three predicted HR. Thus, changes in HR were established by different DiaD (Table 1), which is significantly more influenced by HR^[27,28]^.

Regarding the data acquisition system, the flow and pressure analog inputs were established, respectively, with first order lowpass filters (Butterworth) of 10 Hz and 20 Hz^[12]^. The flow signals were also associated with a median filter of 100 elements, in order to reduce the signal noise from the electromagnetic flowmeter. Sample rate was 1 kHz, based on 1,000 samples.

The arterial compliance was adjusted to near 2.2 mL/mmHg^[9]^ and the peripheral resistance was slightly higher as HR was increased.

## RESULTS

**Fig. 3:**
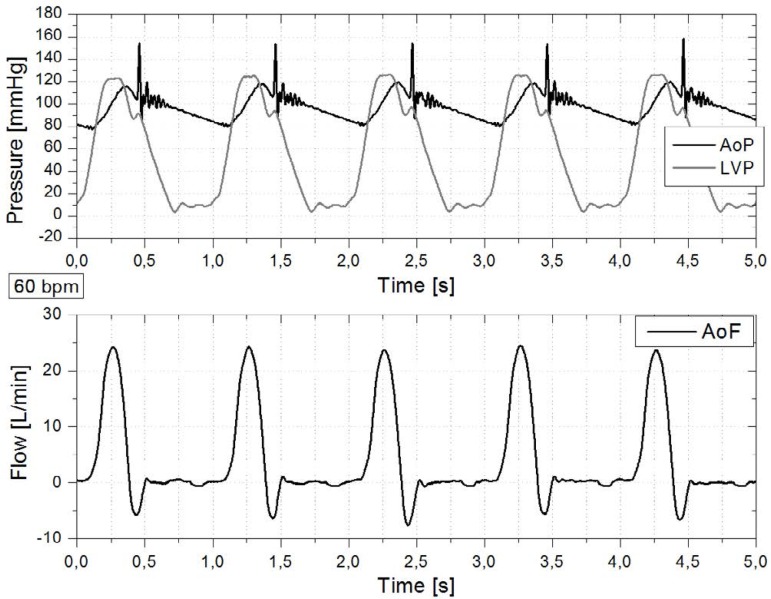
Cardiac simulator signals at 60 bpm. Left ventricular pressure (LVP), aortic pressure (AoP) and flow rate in the aortic root (AoF). The time axis for each waveform starts from the beginning of systole.

**Fig. 4:**
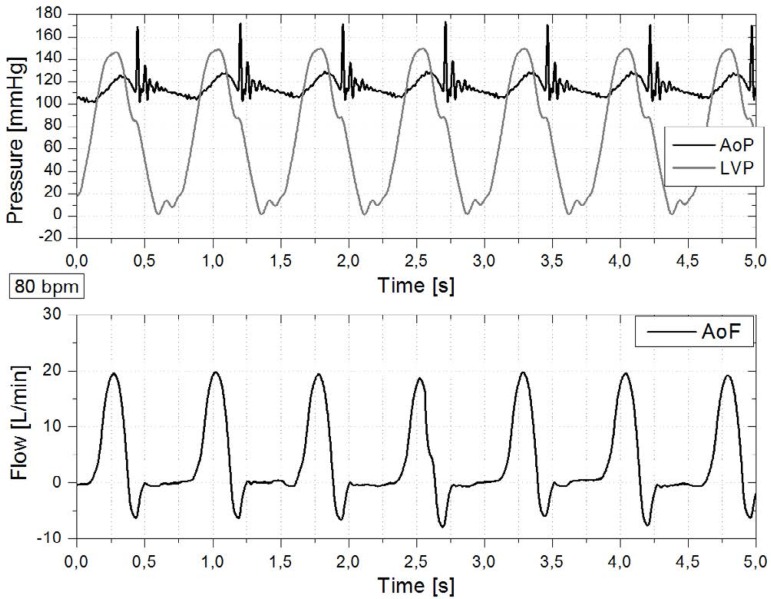
Cardiac simulator signals at 80 bpm. Left ventricular pressure (LVP), aortic pressure (AoP) and flow rate in the aortic root (AoF). The time axis for each waveform starts from the beginning of systole.

**Fig. 5:**
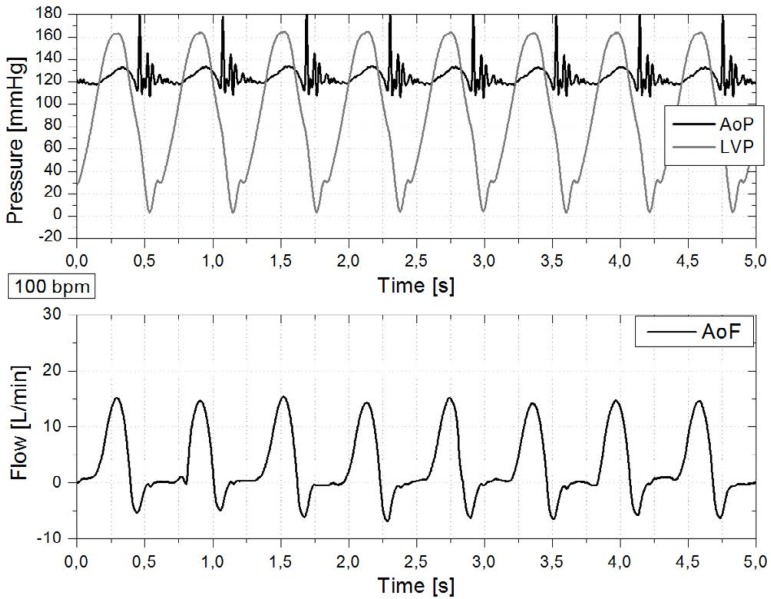
Cardiac simulator signals at 100 bpm. Left ventricular pressure (LVP), aortic pressure (AoP) and flow rate in the aortic root (AoF). The time axis for each waveform starts from the beginning of systole.

Through LabVIEW, waves of LVP, AoP, AoF, and SV were acquired. In order to verify the cardiac simulator ability to replicate some ventricular and systemic circulation characteristics, Figures 3, 4 and 5 were obtained, respectively for 60, 80 and 100 bpm.

All responses from cardiac simulator (according Figures 3 to 5) were inserted in Table 1 for suitable comparison. Afterload was slightly higher as HR was increased, at a constant preload^[40]^.

**Fig. 6:**
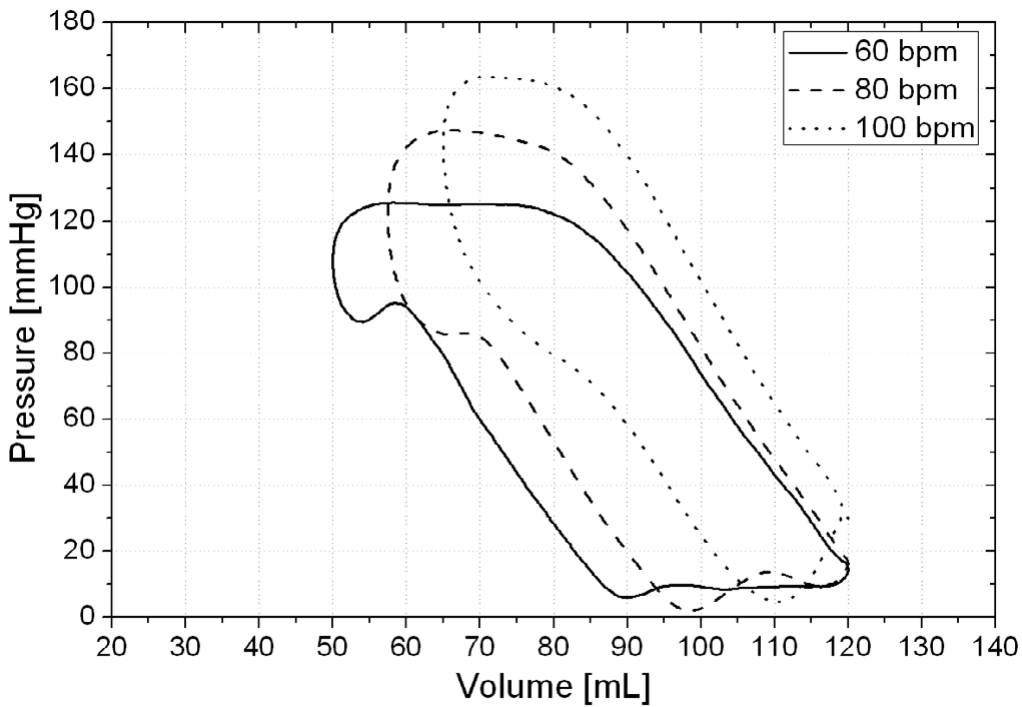
PxV diagrams obtained for different HR (each loop averaged over five consecutive cycles).

LVP *versus* LVV diagram (PxV diagram) were plotted as shown in Figure 6, with each loop averaged over five consecutive cycles.

## DISCUSSION

### Ventricular and Aortic Pressures

Results (Table 1) show that AoP was established according to physiologic parameters^[19,31,33,35]^. In order to allow flow from left ventricular model into the aortic root, the LVP values during systole were slightly higher than the AoP values, for 60 and 80 bpm. Although, for 100 bpm, the LVP rose up to 165 mmHg, that seems normal for elderly population^[32]^. However, all the LVP were consistent according to the literature^[32,39,40]^ and LVP waves detached from the AoP curve during the ejection phase were also observed in some cardiac simulators^[9,10,45]^.

The LVDP oscillated into a normal range for 60 and 80 bpm^[40]^. However, for 100 bpm, the values for the left ventricular end-diastolic pressure (LVEDP) were close to 28 mmHg. Analog results were reported as deficiency in the myocardial relaxation, although with LVEDP values slightly higher^[46]^.

Rapid variations at AoP wave were observed at the instant of the mechanical aortic valve closure, in accordance with the strong impact and the possibility of momentary partial reopening of the leaflets^[5,47]^. At the same time, corresponding wave oscillations were noticed in the LVP shown in Figures 3 and 4, indicating a rapid interruption of these descending pressures. However, it did not appear at 100 bpm (LVP of Figure 5), when the duration of diastole was shorter than at 60 and 80 bpm (see DiaD in Table 1). This means that, although the duration of systole was the same for the three HR, the flow dynamics regarding the isovolumetric relaxation phase (beginning of diastole) at 100 bpm affected differently the AoP wave. As a consequence, the LVP was also different.

### Pressure *Versus* Volume Diagram

According to Figure 6, the PxV diagrams (each loop averaged over five consecutive cycles) demonstrate EDV of 120 mL^[26]^. The values of SV and ESV (Table 1) were similar to some studies including different HR^[19,36]^.

There were no phase in which the pressure increased without changing the intraventricular volume, as would be expected from the isovolumetric contraction (beginning of systole).

In this cardiac simulator, the ventricular filling is not strictly passive, as in projects with a distinct design^[10,11,24]^, but runs according to the hydraulic piston return and flexible membrane deflection - besides the air volumes injected into the work fluid reservoir, like a viscoelastic impedance adapter^[5]^. Anyway, some other cardiac simulators have achieved good results in this sense, regardless of the type of project^[19,20,48,49]^, though not always using a blood analog fluid^[20,48]^.

### Ejection Phase and Cardiac Output

The EPL for all HR was 250 ms. Although the reference value was 210 ms^[31]^, it is possible find *in vitro* studies with EPL up to 300 ms^[22]^. At 60 bpm, the peak flow rate in the aorta was close to 25 L/min, consistent with Dasi et al.^[15]^, in which values of 24 L/min were found. The peak flow rate was lower as HR was increased, according lower values of SV for 80 and 100 bpm. The CO and SV waves were reciprocally consistent as a function of time (and also concerning the LVP data).

The CO was 3.8, 4.2 and 4.9 L/min, respectively for 60, 80 and 100 bpm. These values were lower than expected by theoretical assessment (CO = SV * HR), when aortic regurgitation is neglected. However, the regurgitant volume expected in the aortic bileaflet Carbomedics valve of 27 mm at 70 bpm is about 7.5 mL/beat^[23]^. Thus, the CO results seemed also consistent.

### Other Features

Although the duration of systole was 360 ms, the period from the beginning of the isovolumetric contraction until the end of the isovolumetric relaxation varied depending on HR (Figures 3 to 5). All the values were higher than those mentioned in the literature concerning natural valves, *i.e.*, 320 ms at 70 bpm^[31]^. However, this duration is influenced by the strong impact of the metallic leaflets on the aortic valve closure^[5,47]^.

In the flow through large arteries and heart chambers, blood behaves as a Newtonian fluid, with shear rates higher than 100 sec^-1 [^^[13,37]^. Although, in the vicinity of the hinges - prone to flow disturbances and recirculation -, and where the size of the flow domain is similar to the magnitude of the blood cell size, the non-Newtonian effects must be considered^[14,50]^.

## CONCLUSION

All acquired waves (*i.e.*, LVP, AoP, and AoF responses obtained for 60, 80 and 100 bpm) showed good repeatability for the cardiovascular parameters and prosthetic valves used. Despite some limitations, the cardiac simulator is suitable for *in vitro* evaluation of prosthetic heart valves. Hence, the cardiac simulator was validated to these conditions, in accordance with the human physiological parameters.

Hydrodynamic testing of prosthetic heart valves can be started, once the cardiac simulator operating parameters allow valid experimental comparisons of flow through mitral or aortic prostheses.

### Future Studies

Since the optical accesses were provided in the ventricular model, it is possible to apply, in future works, the PIV and LDA systems^[13,15,23]^. These results can be used to obtain a computational model of the flow^[14,15]^. Further studies may also consider a development of a full closed-loop control for the simulator, where its responses should be evaluated by the PxV diagram for several physiological conditions dynamically^[11,19,20,48,49,51]^.

### Limitations

Some limitations of the cardiac simulator are inherent to the *in vitro* condition. The left ventricular model is not completely flexible and cannot simulate the twisting motion that occurs in the human heart. Furthermore, the ventricular filling is not passive. These conditions may affect the LVP, AoP and CO waves, for instance, which were also attenuated by the low-pass filters used (cut-off frequencies of 20 Hz for LVP and AoP, and 10 Hz for CO).

Some well-known variables from the human physiological literature (regarding the natural heart valves) served as parameters for this validation experiment. However, they were not strictly met, since cardiac prostheses were used.

In this work, we did not satisfy the requirements from the ISO 5840 concerning the report of the prosthetic valves behavior or the analysis of their hydrodynamic performance, as the quantification of both effective orifice area and regurgitant volumes^[12]^.

**Table:** 

Authors' roles & responsibilities	
OB	Analysis and/or data interpretation; conception and design study; manuscript redaction or critical review of its content; realization of operations and/or trials; final manuscript approval
JPO	Analysis and/or data interpretation; conception and design study; manuscript redaction or critical review of its content; final manuscript approval
